# Is sperm telomere length altered in teratozoospermia specimens? A case-control study

**DOI:** 10.18502/ijrm.v21i3.13198

**Published:** 2023-04-14

**Authors:** Maryam Fattahi, Mohadese Maghsudlu, Mohammad Hasan Sheikhha

**Affiliations:** ^1^Department of Medical Genetics, Shahid Sadoughi University of Medical Sciences, Yazd, Iran.; ^2^Department of Medical Genetics, School of Medicine, Tehran University of Medical Sciences, Tehran, Iran.; ^3^Abortion Research Center, Yazd Reproductive Sciences Institute, Shahid Sadoughi University of Medical Sciences, Yazd, Iran.

**Keywords:** Telomere, Teratozoospermia, Sperm, Male infertility.

## Abstract

**Background:**

Male factor infertility is a multifactorial defect, and many of its etiologies are unknown. Teratozoospermia is determined by the existence of over 85% morphologically abnormal spermatozoa in semen which are almost incompetent in fertilization function. One of the most novel issues in genetic alterations studies is the variation of sperm telomere lengths (STL) and its collaboration with male infertility. The present study has been focused on STL alterations in teratozoospermia.

**Objective:**

Investigation of differences in telomere length of teratozoospermia specimens and sperms with normal parameters.

**Materials and Methods:**

In this case-control study, 60 men referred to Arak Fertility Clinic, Markazi province, Iran from November 2017 to February 2018 were categorized into teratozoospermia and normozoospermic groups. Sperm genomic DNA extraction was conducted, and STL were evaluated using quantitative polymerase chain reaction.

**Results:**

Statistical evaluation of relative telomere length was calculated by the ratio of telomere to single-copy gene for teratozoospermia and normal specimens. Results significantly demonstrated that relative telomere length in teratozoospermia samples is nearly 3 times shorter than in normal samples (p > 0.001).

**Conclusion:**

Our results represent the reduction of telomeres length in teratozoospermia and suggest that this alteration might be one of the factors contributing to the sperm fertility potential of this kind of specimen. However, defining relevant molecular processes requires further detailed investigations.

## 1. Introduction

Fertility is one of the major challenges in the modern era and afflicts almost 8-12% of couples worldwide, with even higher prevalence in industrialized countries (1, 2). Male infertility is responsible for 40-50% of these cases, and the percentage of infertile men is approximately 2.5-12% worldwide (1). Infertility of male origin has numerous and multifactorial causes such as anatomic, endocrine, metabolic, and genetic problems, which in some cases can lead to numerical and morphological disorders of sperms and low semen quality (3). Among these abnormalities, teratozoospermia is described by the presence of more than 85% spermatozoa with irregular morphology in semen (4, 5). This heterogeneous category includes a broad range of sperm phenotypes that affect structures of the spermatozoa (6). It seems sperm maturation in testis is defected in teratozoospermia patients and abnormal sperms are functionally incompetent. In vitro fertilization and intracytoplasmic sperm injection are useful methods for obtaining live births in patients with teratozoospermia. Although the outcomes seem poor in most cases, thus clarifying molecular aspects of this defect is of great importance (7).

Various causes such as abnormal sperm parameters and sperm DNA damage are known to decrease the chance of fertility. Lately, studies in reproductive fields have increasingly focused on telomere length (8, 9). Telomeres are composed of single-stranded DNA and DNA-protein complexes located at the end of chromosomes and are well known for their protective role against chromosome degradation and fusion. The single-stranded DNA is a noncoding tandem repeat sequence with hexa nucleotide 5 ' -TTAGGG-3 ' , which play a key role in maintaining chromosomal stability and cell viability (10, 11). This sequence extends for 5-10 kilobases in human somatic cells, while in male germ cells, the average length is doubled. Although the length of telomeres decreases with each cell division, it has been observed that in male germ cells, the length of telomeres increases with age indicating the importance of preserving genomic integrity between generations (12, 13).

Over recent years, telomere biology has attracted significant attention in human reproduction, leading researchers to focus on the relation between sperm telomere length (STL) and male fertility and spermatogenesis. The studies assessing telomere length in sperms suggest that in low-quality semen samples and even in infertile men with normal semen indices, the telomere is shortened compared to fertile men (14). Studies show that reduced telomere length in spermatozoa may be a marker of abnormal spermatogenesis, which can represent feasible problems (15, 16).

The direct relation between teratozoospermia and telomere length had not been evaluated in previous studies. Thus, this study aimed to assess any differences between telomere length with normal and teratozoospermia sperm in men of the same age range.

## 2. Materials and Methods

### Study population

In this case-control study conducted from November 2017 to February 2018, semen samples (n = 30) were taken from infertile men attending Arak Fertility Clinic, Markazi province, Iran. Teratozoospermia has been approved by specialists considering the result of semen analysis (including sperm count, sperm morphology, semen density, motility grade, etc.). The participants included were aged under 40 with no history of varicocele, infection, chronic illness, chemotherapy, radiotherapy, or drug addiction.

In addition, 30 healthy donors with proven normal semen profiles were used as controls. Case and control groups were similar in terms of age and absence of illnesses and therapies.

### Genomic DNA extraction and quantitative polymerase chain reaction (qPCR)

Genomic DNA was extracted directly from semen specimens using the GeneAll kit (General Biosystems, Seoul, Korea). Almost 100 µl of semen was used for extraction according to the manufacturer's instructions. Quality assessment was then performed by measuring the concentration and purity of extracted DNA.

STL was assessed using qPCR, according to Cawthon. In this method the signal intensity received during qPCR was used to quantify the amount of telomeric DNA as copy number variation relative to the amount of a single copy gene which result in a ratio called as telomere/single copy gene (T/S) ratio. The *36B4* gene that encodes acidic ribosomal phosphoprotein was used as the single-copy reference gene (17).

All samples were run in duplicate along with a negative control included in each run. The reaction well contained 25 ng DNA and 15 µl master mix 2X invitrogen (Applied Biosystems, USA), 2 pairs of primers (forward primer and universal reverse primer), and water to a final volume of 30 µl. The sequence of primers used in qPCR is included in table I. The ABI step one plus RT-PCR (Applied Biosystems, USA) was used for running the test, and the thermal cycling program was as follows: 10 min at 95 C, and 40 cycles of 95 C for 15 min and 60 C for 1 min, 10 min at 95 C and 35 cycle of 15 sec at 95 C and 30 sec at 56 C and 1 min at 72 C.

**Table 1:** Sequence of primers used in qPCR


**Primer**	**Length**	**Sequence**
**Tel-F**	37	GGTTTTTGAGGGTGAGGGTGAGGGTGAGGGTGAGGGT
**Tel-R**	37	TCCCGACTATCCCTATCCCTATCCCTATCCCTATCCCTA
**36B4-F**	25	CCCATTCTATCATCAACGGGTACAA
**36B4-R**	23	CAGCAAGTGGGAAGGTGTAATCC
qPCR: Quantitative polymerase chain reaction, Tel-F: Forward primer telomere, Tel-R: Reverse primer for telomere, 36B4-F: Forward primer for 36B4, 36B4-R: Reverse primer for 36B4		

### Ethical considerations

The informed consent was accepted by all contributors, and the study was approved by the Ethics Committee of Shahid Sadoughi University of Medical Sciences, Yazd, Iran (Code: IR.SSU.MEDICINE.REC.1396.145).

### Statistical analysis

Data are provided as mean ± standard deviation using GraphPad Prism6 (GraphPad Software Inc., San Diego, USA) and SPSS (SPSS Inc., Chicago, IL) software. To access the parametric nature of data, the Shapiro-Wilk normality test was applied. Since the normality test results showed that the data was parametric (p < 0.05), a parametric *t* test was used to evaluate the differences between the teratozoospermia and normal specimens, and p < 0.05 was considered statistically significant.

## 3. Results

**Figure 1:**
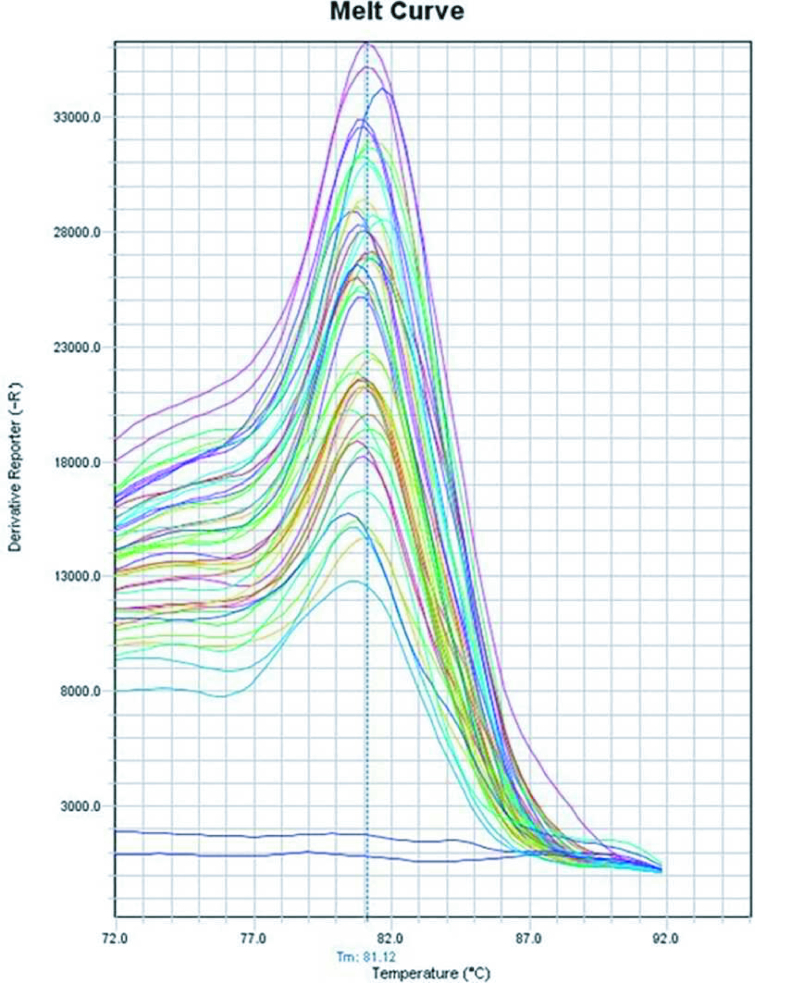
Melt curve of qPCR showed sharp and singular peaks which confirms the specific amplification and absence of contamination.

Q-PCR curves were evaluated to verify the accuracy of the test, and melt curves showed sharp and singular peaks while negative controls had not shown any amplification (Figure 1).

**Figure 2:**
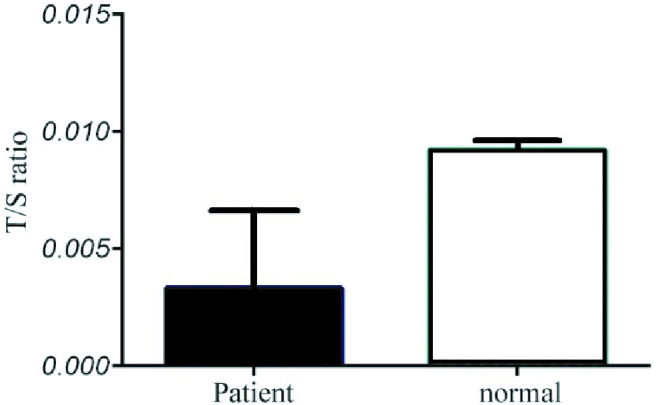
The ratio of telomere to singular gene in the normal and patient groups.

To evaluate the relative telomere length, the T/S ratio was calculated for each specimen using the 2 ${}^{-\Delta \Delta Ct}$ formula. The lowest and highest amount of T/S ratio calculated for teratozoospermia samples were respectively 0.0001 and 0.0180 with a mean of 0.0033 (SD = 0.0034), while the T/S ratio for normal samples was calculated between 0.0082 and 0.0171 with a mean of 0.011 (SD = 0.0029). The results indicated that relative telomere length in normal samples was nearly 3 times higher than in teratozoospermia samples (Figure 2). In order to check if the difference between 2 tested groups is significant or not, the comparison of the T/S ratio among case and control groups was made by unpaired parametric *t* test, and the results were significant by p < 0.001.

## 4. Discussion

In the present study, relative telomere length of teratozoospermia patients were compared with standard samples and the results indicated that telomere lengths of teratospermic specimens were significantly shortened. This alteration might be one factor contributing incompetency of this kind of sperms. To our knowledge, teratospermic specimens were not evaluated separately but were included in some studies as a part of an abnormal group (18). Several studies have investigated the relationship between telomere length and male infertility and mostly reported the suggestive relationship between STL and male infertility (16, 19, 20). However, there is a contradiction between studies regarding the correlation between telomere length of sperm and abnormal sperm parameters. A previous study comparing oligozoospermic and normozoospermic specimens have reported that patients with oligozoospermia mostly display shortened STL along with high sperm DNA fragmentation. Moreover, viable pregnancies were not achieved when samples with atypical STL were used during assisted reproductive technology (21). While the results of another study by Berneau et al. on normozoospermic infertile men indicated no association between STL and sperm parameters such as sperm count, or progressive motility (22). The study by Liu et al., comparing men with idiopathic infertility and fertile men indicated an association between shorter telomere length and infertility, along with a relationship between the telomeres length and quantity and motility of sperms (23). Another study, conducted on sub-fertile men, reported that telomere hemostasis is significantly impaired among specimens of sub-fertile sperms and that shorter telomere length is observed in them (24).

Overall, a recent review states that the effect size of STL on fertility parameters was relatively `medium'; however, further investigation is suggested to ascertain whether examining the telomere length of sperms can be useful as a predictive clinical test for male factor infertility (25).

Infertility as a reproductive health problem has been growing in recent decades and affects nearly 8-12% of couples at the reproduction age (1). Male factor involvement accounts for half of the infertility issues (26). Since the contingency of conception depends on semen quality, evaluating semen parameters is the basic diagnostic method for infertile men; however, these procedures are not efficient enough in explaining the causes of the disorder (27). One of the categories regarding preliminary classifications of main male infertility causes based on semen analysis includes teratozoospermia, which mostly indicates a flaw in sperm maturation in the testis and can be divided into 2 subgroups, monomorphic teratozoospermia in which all the sperms from an ejaculation have the same deformity, and polymorphic teratozoospermia that shows a verity of malformations in ejaculation (28, 29). Various factors are responsible for this abnormality, such as, hormonal disorders, varicocele, exposure to spermatozoids, and genetic issues (4, 7).

Genetic alterations are known as remarkable actors among the different biological factors that affect semen characteristics (30). Recently, telomeres, nucleoprotein structures protecting the end of eukaryotic chromosomes, have been in the spotlight. These complex structures, characterized by repetitive DNA sequences of 5 ' -TTAGG-3 ' , have the mission to stabilize chromosomes and prevent them from degradation or fusion and ensure the occurrence of qualified recombination (31-33). These protective construction decreases in length during each cell division, which is directly related to the aging process.

Moreover, in male germ cells, telomere length does not only shorten but also increases to avoid transmission of shortened telomeres to the next generation (34). Telomere structure and length have a significant role in genomic integrity conservation. Besides, it has crucial functions in synapsis and recombination during meiosis (35). Moreover, studies have reported that at the time of spermatogenesis, telomeres migrate toward and associate with the nuclear membrane, which is believed to play a critical role in pronucleus formation and early development (36, 37). Considering the issues raised, it can be inferred that sperms with shorter telomere lengths may not perform well in fertilization and blastocyst formation (12).

Considering STL as an additional sperm parameter, it might widen new perspectives for management of infertile couples. Reduced telomere length may be one of the responsible factors for male infertility, but further detailed investigations are required to stabilize this viewpoint.

## 5. Conclusions

Amongst different biological factors that affect semen characteristics, genetic alterations are known as remarkable actors, and telomeres length is one of the issues which has been highlighted in this field lately. Our results represent the reduction of telomeres length in teratozoospermia and suggest that this alteration might be one of the factors contributing to the sperm fertility potential of this kind of specimens. However, defining relevant molecular processes requires further detailed investigations.

##  Conflict of Interest 

The authors declare that they have no competing interest.
